# Postoperative spinal orthosis may not be necessary for minimally invasive lumbar spine fusion surgery: a prospective randomized controlled trial

**DOI:** 10.1186/s12891-021-04490-4

**Published:** 2021-07-12

**Authors:** Hsuan-Hsiao Ma, Pei-Hsi Wu, Yu-Cheng Yao, Po-Hsin Chou, Hsi-Hsien Lin, Shih-Tien Wang, Ming-Chau Chang

**Affiliations:** 1 Department, of Orthopedics and Traumatology, Taipei Veterans General Hospital, No. 201, Section. 2, Shi-pai Road, Taipei, 11217 Taiwan, ROC; 2 School, of Medicine, National Yang Ming Chiao Tung University University, Taipei City, Taiwan, ROC

**Keywords:** Brace, Clinical Outcome, Minimally invasive surgery, Radiographic solid fusion, Lumbar spinal orthosis, TLIF

## Abstract

**Background:**

With the progress and success in minimally invasive surgery of transforaminal lumbar interbody fusion (MIS TLIF), the musculoskeletal injury was minimized. However, the role of postoperative orthosis in MIS TLIF has not been established and there is little evidence supporting the routine use of orthosis in MIS TLIF.

**Methods:**

This is a prospective randomized clinical study. 90 patients who underwent MIS TLIF were randomly divided into groups A (with postoperative spinal orthosis) and B (without postoperative spinal orthosis). Patients were followed up for an average of 12.6 months. Clinical outcome was assessed using the Oswestry disability index (ODI) and visual analogue scale (VAS). Fusion rate was classified with the BSF scale system at postoperative 6-month, and 12-month.

**Results:**

Both groups had similar patient demographics. The use of postoperative spinal orthosis had no significant influence on instrumentation-related complications or radiological parameters at each follow-up.

**Conclusions:**

In this study, we conclude that postoperative spinal orthosis is not necessary for MIS TLIF. Patients without postoperative spinal orthosis had the same fusion rates and improvement of VAS and ODI scores.

## Background

Lumbar orthoses are used extensively in various conditions including low back pain [1], spondylolisthesis, spinal deformity, and during the postoperative period [2]. However, the necessity of rigid bracing after lumbar fusions has been questioned. In general, immobilization of any musculoskeletal injury will help in the relief of the pain secondary to the injury [3]. And the goals of postoperative orthosis include immobilizing gross body motion [4] and motion segments, unloading the force [5], reducing pain [6], enhancing the fusion rate [7], and improving functional outcomes [8].

The effect of restricting gross motions of the trunk rather than intervertebral mobility by orthosis in the lumbar spine was confirmed using roentgen stereophotogrammetric analysis [9]. Besides, the functional outcomes were found not improved by postoperative bracing in posterior [10] and posterolateral spinal instrumented fusion [11]. Moreover, the drawbacks of postoperative orthosis include muscle atrophy [12], skin irritation, additional costs, and delays in rehabilitation.

With the progress and success in minimally invasive surgery of transforaminal lumbar interbody fusion (MIS TLIF), the musculoskeletal injury was minimized with significant reductions of operative time, length of stay, VAS scores compared with the open technique [13]. However, the role of postoperative orthosis in MIS TLIF has not been established and there is little evidence supporting the routine use of orthosis in MIS TLIF. Thus, we performed a prospective randomized trial to evaluate the necessity of a postoperative external lumbar orthosis as an adjunct therapy for patients treated with MIS TLIF for degenerative conditions.

## Methods

Between November 2016 and April 2017, 90 patients who underwent MIS TLIF in our institute were enrolled prospectively after institutional review board approval (2015–08-006ACF) and the protocol has been registered in ClinicalTrials.gov. Indications for surgery were grade 1 spondylolisthesis, pars fractures, and degenerative discs with back pain or radiculopathy that involves only one or two segments. The surgical level ranges from the second lumbar vertebra (L2) to the first sacral vertebra (S1). All surgeries were performed by a single surgeon. Patients with spinal fracture, spinal infection, intraspinal tumor, previous spine surgery, osteoporosis (T < -2.5), systemic autoimmune disease, end-stage renal disease, and Parkinsonism were excluded.

### Surgical technique

Under general anesthesia, patient was placed in prone position on the four-poster frame. Intraoperative 3-dimensional C-arm (Vision FD Vario 3D, Ziehm Imaging, Nuremberg, Germany) and navigation system (Kick navigation system, Brainlab) were used for percutaneous pedicle screw insertions. Intraoperative scanning was performed in both groups with CT data set automatically registered to the image guidance system. Before the 1.5-cm skin incision was made, the spinal process was used as an anatomical landmark by navigation probe to confirm the accuracy of navigation. The ideal skin entry points were sought with the navigation probe tip in order to determine the precise skin incision based on the virtual elongated navigation trajectory on the intraoperative scanned images. Pedicle entry points were decided on navigation guidance, and pilot holes were made with a 3-mm awl. The direction of the pilot pedicle hole was rechecked with the navigation probe, and 4.5-mm tapping was done to a depth of 20 mm from the entry point. A guidewire was used for screw insertion. The size of the pedicle screws was determined under the navigation system. Rod was then placed and fixed with nuts after rechecking the position. Microscopic decompression and transforaminal lumbar interbody fusion was then performed under the incision for pedicle screws with cage, demineralized bone matrix(OsteoSelect AQ3 DBM Putty, Bacterin International Inc., Belgrade, MT), and autologous bone graft [14, 15]. All surgeries were performed by 1 senior spinal surgeon.

### Postoperative care

**Fig. 1:**
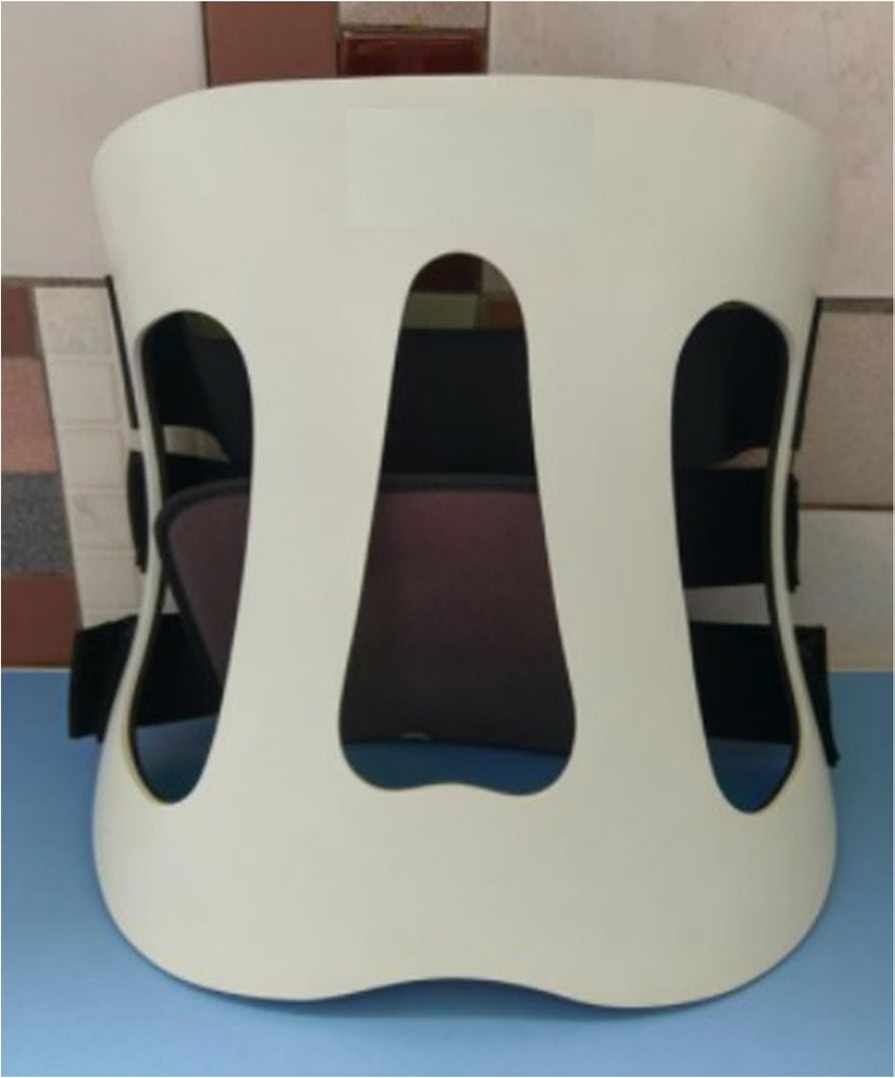
The Rigid chairback brace used in this study

**Fig. 2:**
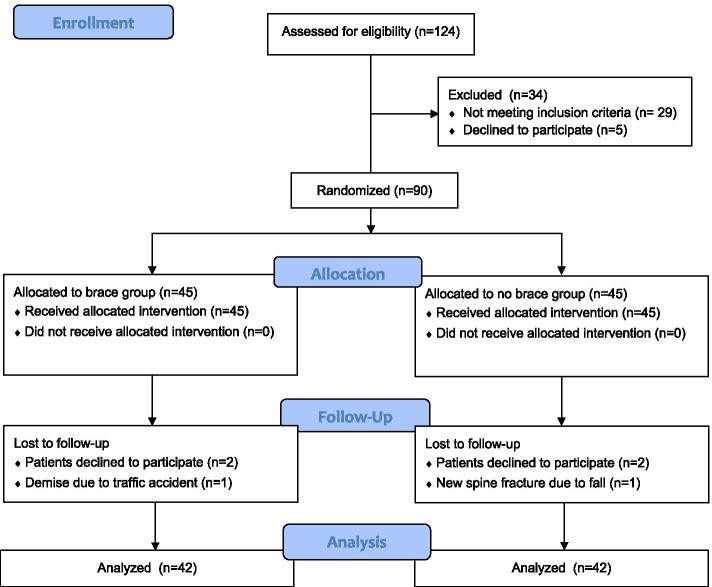
Consort diagram

They were randomly distributed to either group A or group B using computer-generated random numbers. Patients in group A undergone MIS TLIF were protected with postoperative spinal orthosis for 3 months. A rigid chairback brace (Fig. 1) was used full-time after ambulation except bathing and lying on bed. Patients in group B did not use any rigid brace or lumbar corset. The allocation was shown in the consort-diagram (Fig. 2).

### Clinical evaluation and functional assessment

**Fig. 3:**
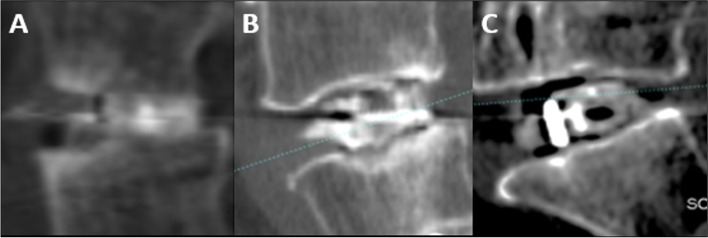
**A** BSF-3, fusion with remodeling and trabecular present. **B** BSF-2, graft intact, not fully remodeled and incorporated, but no lucency present. **C** BSF-1, graft intact but with lucency visible around the graft or cage

Patients were followed at postoperative 1-month, 3-month, 6-month, and 12-month. Radiographs included anteroposterior, lateral lumbar spine views, and lateral standing flexion–extension views. The success of interbody fusion was evaluated by computed tomography (CT) at the 6-month and 12-month postoperative follow-up using the Brantigan-Steffee-Fraser (BSF) classification. An additional computed tomography (CT) scan was done in cases of uncertainty of screw position or in in case of symptomatic patients during the follow up. Clinical and radiographic assessments were performed by an independent blinded observer not involved in patient care. As seen radiographically, fusion was graded by BSF classification (Fig. 3).

Perioperative parameters were recorded including surgical duration, timing of ambulation, length of hospital stay and complications. All patients were regularly followed up at our clinics. There were 1 patient in each group had accidental falling and were excluded. None of the patients dropped out of the study due to unknown reasons. Clinical outcomes were assessed using the Oswestry disability index (ODI) and visual analogue scale (VAS) preoperatively, at 1-, 3-, 6-, and 12-month postoperatively.

### Statistical analysis

To determine the ideal sample size required to achieve an alpha level of 0.05 with 90% power, we assumed noninferiority of “without brace” compared with “with brace” if the mean difference of the ODI score across interventions was less than one-third of minimal clinical important difference (MCID). The MCID of the ODI based on the previous study was 14.9 points and the estimated standard deviation of 11.3 [16, 17]. Assuming a 20% loss to follow-up, a final ideal sample size of at least 30 patients in each group was determined.

The patients’ age, body mass index (BMI), preoperative VAS, preoperative ODI, operative time, timing of ambulation and length of stay were analysed with independent sample t test. The clinical outcomes (VAS and ODI) of each group after operation were analysed using paired t test. Categorical data were compared using the χ2 or the Fisher exact test. Differences in sex, medical history and complications between groups were assessed using Mann–Whitney Test. Statistical significance was set at *p* < 0.05. All statistical analyses were performed using SPSS version 20 (IBM, Chicago, IL, USA).

## Results

The mean age of the patients was 69.1 ± 9.1 years (range, 48–78 years) in group A and 70.8 ± 8.9 years (range, 56–85 years) group B. The mean BMI of the patients was 26.2 kg/m^2^ in group A and 26.1 kg/m^2^ in group B. In group A, 30 and 12 patients underwent one- and two-level surgery, respectively, whereas in group B, 30 and 12 patients underwent one- and two-level surgery, respectively.

In group A, 6 patients had diabetes mellitus and 7 patients were smokers. In group B, 12 patients had diabetes mellitus, and 2 patients were smokers. Age, sex, BMI, levels of surgery, underlying medical condition (diabetes mellitus and smoking) showed no significant difference between the two groups (Table 1).

**Table 1:** Patient demographics

	Group A (*n* = 42)	Group B (*n* = 42)	*p-value*
Age (years, mean ± SD)	69.1 ± 9.1	70.8 ± 8.9	0.645
Sex (female, %)	34 (81.0%)	30 (71.4%)	0.443
BMI (kg/m^2^, mean ± SD)	26.2 ± 3.2	26.1 ± 3.6	0.985
DEXA (T-score, mean ± SD)	-1.0 ± 1.52	-1.1 ± 1.18	0.780
Spinal level, fused (n, %)			0.715
L2-3	0 (0)	2 (4.8)	
L3-4	2 (4.8)	3 (7.1)	
L3-L5	6 (14.3)	7 (16.7)	
L4-5	26 (61.9)	22 (52.4)	
L4-S1	6 (14.3)	5 (11.9)	
L5-S1	2 (4.8)	3 (7.1)	
Medical history (n, %)			
DM	6 (14.2)	12 (28.6)	0.183
Smoking	7 (16.7)	2 (4.8)	0.156

*BMI* body mass index, *DM* diabetes mellitus

The preoperative VAS is 5.9 ± 1.4 in group A and 5.6 ± 0.8 in group B, and the preoperative ODI is 51.0 ± 13.9 in group A and 48.3 ± 10.6 in group B. There were no significant differences in preoperative VAS (*p* = 0.181) and ODI (*p* = 0.469) between the two groups (Table 1). The mean operative time was 3.9 h in group A, and 4.2 h in group B. There was no significant difference between each group (*p* = 0.480). The timing of ambulation is 2.2 days in group A and 2.0 days in group B. The length of hospitalization is 7.0 days in group A and 6.9 days in group B (*p* = 0.588) (Table 2).

**Table 2:** Perioperative and clinical outcomes

	Group A (*n* = 42)	Group B (*n* = 42)	*p*
Operative time (hours, mean ± SD))	3.9 ± 1.02	4.2 ± 1.05	0.480
Timing of ambulation (days, (mean ± SD)	2.2 ± 1.22	2.0 ± 1.12	0.220
Length of stay (days, (mean ± SD)	7.0 ± 2.23	6.9 ± 1.98	0.588
VAS (mean ± SD)			
Preoperative	5.9 ± 1.4	5.6 ± 0.8	0.181
Postoperative 1 months	1.4 ± 1.30	1.3 ± 0.97	0.923
Postoperative 3 months	1.30 ± 1.13	1.2 ± 0.96	0.823
Postoperative 6 months	1.4 ± 1.12	1.1 ± 0.57	0.259
Postoperative 12 months	1.2 ± 1.46	1.1 ± 1.18	0.882
ODI (mean ± SD)			
Preoperative	51.0 ± 13.9	48.3 ± 10.6	0.469
Postoperative 1 month	18.0 ± 4.42	18.8 ± 10.11	0.764
Postoperative 3 months	14.0 ± 9.40	13.2 ± 6.16	0.777
Postoperative 6 months	10.3 ± 4.96	10.2 ± 5.35	0.982
Postoperative 12 months	8.6 ± 4.90	8.2 ± 5.63	0.898

*VAS* Visual Analogue Scale, *ODI* Oswestry disability index

The VAS score at 1 month was 1.4 ± 1.30 in group A and 1.3 ± 0.97 in group B, and the VAS scores at 12 months were 1.2 ± 1.46 in group A and 1.1 ± 1.18 in group B. The ODI score at 1 month was 18.0 ± 4.42 in group A and 18.8 ± 10.11 in group B, whereas the ODI scores at 12 months were 8.6 ± 4.90 in group A and 8.2 ± 5.63 in group B. VAS and ODI scores significantly improved after surgery in both groups. The amplitude of VAS and ODI improvement was similar at each follow-up between both groups.

For the purposes of statistical analysis, BSF-2 and BSF-3 on CT scans were regarded as successful fusion, and BSF-1 was regarded as non-union. In the entire cohort, there was no BSF-1 in each group. The fusion rates at 6-month and 12-month follow-up showed no significant between the groups (Table 3).

**Table 3:** Fusion rate between two groups

	Group A (*n* = 42)	Group B (*n* = 42)	*P* value
BSF classification by CT scan (n, %)			1.000
BSF-1	0 (0%)	0 (0%)	
BSF-2	3 (7.1%)	2 (4.8%)	
BSF-3	39 (92.9%)	40 (95.2%)	
Fusion rate (%)			
6-month	83%	81%	0.928
12-month	100%	100.0%	

*BSF* Brantigan-Steffee-Fraser

In terms of complications, there were one superficial wound infection and one pedicle screw loosening in group A and one early postoperative pulmonary embolism in group B which totally recovered after medical treatment. Overall, complications occurred in 4.8 and 2.4% of the patients in the control and experimental group respectively (Table 4).

**Table 4:** Complications and reoperations

	Group A (*N* = 42)	Group B (*N* = 42)	*P* value
Complications			-
Perioperative complications			-
Superficial wound infection	1	0	-
Epidural hematoma	0	0	-
Nerve root damage	0	0	-
Implants dislodge	0	0	-
Medical-related complications			-
Pulmonary embolism	0	1	-
Implants-related complications			-
Screw loosening	1	0	-
Screw broken	0	0	-
Cage migration	0	0	-
Total (n)	2	1	1.000
Reoperation due to complications	0	0	-

All data were expressed as number of patients

## Discussion

Historically, the use of a spinal orthosis after open lumbar fusion surgery is generally adopted. However, earlier studies revealed insufficient evidence that lumbar supports are more effective than no treatment [8]. And there is an obvious lack of consensus regarding the most appropriate type, duration, and indications for immobilization that support the routine use of postoperative lumbar supports after spinal surgeries [18]. For thoracolumbar fractures, treatment using early ambulation without a brace avoids the cost and patient deconditioning associated with a brace [19], and the Oswestry Disability Index scores for the treatment of compression fractures without a brace were not inferior to those with soft or rigid braces [20].

For lumbar spine fusion surgery, the benefits of using postoperative brace are controversial. There was no significant impact on the risk of non-union following cervical or lumbar fusions [21]. Despite no negative effect by the continuous use of a lumbosacral orthosis for 1–6 months, there might be the influence of duration of postoperative lumbar immobilization with the aid of a rigid lumbar orthosis on the consolidation of posterolateral lumbosacral fusions [22]. However, the quality of evidence ranged from low to very low in one meta-analyses study [23]. And the routine use of bracing following instrumented posterolateral fusion is not recommended [24].

The MIS technique preserves the posterior tension band, and the back muscle groups were minimally detached. However, transforaminal technique always includes unilateral or bilateral facetectomy [25], and removal of the intervertebral disc, which may cause greater instability than performing posterolateral fusion. In our study, all patients received unilateral facetectomy, and there was no significant difference in fusion assessment between two groups at 3-month, 6-month, and 12-month follow-up. Although a CT scan may show a high sensitivity for pseudarthrosis compared with plain film, plain X-ray films and helical CT scans showed equal accuracy after posterior lumbar interbody fusion confirmed by surgical exploration [26].

Patients might feel more comfortable and it might be more convenient for earlier postoperative rehabilitation without a postoperative spinal orthosis. We designed this study to assess the bracing effect on patient-derived functional outcomes. In our experience, patients feel discomfort when putting-on or taking-off the brace, which may make them hesitate from starting mobilization.

This study has some limitations. Osteoporotic patients were excluded as non-union and cage subsidence are well-known complications in osteoporotic patients who undergo lumbar interbody fusion surgery [27]. Minor subsidence might be due to end-plate manipulation during cage insertion and was not included in the complications in this study. Future studies of postoperative bracing in osteoporotic patients may be needed. Second, the fusion rate at 12-month seems to be too high and postoperative ODI seems to be lower in both groups. The reasons might include small sample size with the same experienced surgical technique, routine DBM use, and cultural response in Taiwan. Third, minor complications, such as durotomy, urinary tract infection, and persistent neurological paresthesia were not reported in this study. Forth**,** brace compliance in clinical scenario lacks a reliable and objective measure since clear instructions about brace wear were given.

To our best knowledge, this study is the first prospective randomized trial to evaluate the outcomes both functionally and radiographically following minimally invasive transforaminal lumbar interbody fusion for degenerative conditions. We believed that not using a postoperative orthosis would achieve a more comfortable postoperative period, easier recovery and higher patient satisfaction.

## Conclusion

The necessity of routinely used postoperative spinal orthosis in lumbar spine surgery has been questioned. Previous studies found no benefits in the use of orthosis in reducing pain or preventing non-union. In this study, we found that postoperative spinal orthosis does not improve outcomes in MIS TLIF. Patients without postoperative spinal orthosis had the same fusion rates and improvement of VAS and ODI scores.

## Data Availability

The information to access the data used in the study is included within this article.
